# Single and combined associations of blood lead and essential metals with serum lipid profiles in community-dwelling adults

**DOI:** 10.3389/fnut.2023.1129169

**Published:** 2023-04-14

**Authors:** Heng Wan, Dongmei Wang, Yongqian Liang, Yajun He, Qintao Ma, Tingting Li, Yingbo He, Hanquan Guo, Jiachen Wang, Zhao Li, Xu Lin, Lan Liu, Jie Shen

**Affiliations:** ^1^Department of Endocrinology and Metabolism, Shunde Hospital, Southern Medical University (The First People's Hospital of Shunde), Foshan, Guangdong, China; ^2^School of Public Health, Southern Medical University, Guangzhou, Guangdong, China; ^3^Department of Business Development, Shunde Hospital, Southern Medical University (The First People's Hospital of Shunde), Foshan, Guangdong, China

**Keywords:** lead, magnesium, lipid profiles, metal mixture, Bayesian kernel machine regression

## Abstract

**Background:**

Although several studies have examined the relationships between lead (Pb) exposure and serum lipid profiles, the associations of the metal mixture, including lead (Pb) and essential metals with lipid profiles, remain unclear.

**Objective:**

To investigate the associations of the metal mixture including Pb and essential metals [magnesium (Mg), manganese (Mn), copper (Cu), iron (Fe), zinc (Zn), and calcium (Ca)] with serum lipid profiles [total cholesterol (TC), triglyceride (TG), low-density lipoprotein cholesterol (LDL-C), and high-density lipoprotein cholesterol (HDL-C)], as well as the potential interactions among the metals.

**Methods:**

Nine hundred and ninety-eight Chinese community-dwelling adults completed a questionnaire and underwent checkups of anthropometric parameters, serum lipid profile levels (TC, TG, LDL-C, and HDL-C), and blood metal concentrations (Pb, Mg, Mn, Cu, Fe, Zn, and Ca). The multivariable linear regression, weighted quantile sum (WQS) regression, and Bayesian kernel machine regression (BKMR) were applied to evaluate the single and combined associations of blood Pb and essential metals with serum lipid profiles.

**Results:**

In the multivariable linear regression model, the blood Pb was positively associated with TC, LDL-C, and HDL-C (*p* < 0.05, all), and the blood Mg were positively associated with serum TC, LDL-C, and Ln TG (*p* < 0.05, all). In the WQS regression and BKMR models, the metal mixture of blood Pb and the essential metals was positively associated with all of the serum lipid profiles. In addition, an inverse U-shaped association of Pb with Ln TG and the positive interactive effect between blood Pb and Mg levels on TC and LDL-C were found.

**Conclusion:**

The levels of blood Pb, together with the essential metals, especially Mg levels, are suggested to be considered when assessing dyslipidemia risk. However, more evidence is still needed to validate the conclusions.

## Introduction

Dyslipidemia is characterized by an imbalance of lipid profiles, including higher levels of total cholesterol (TC), triglyceride (TG), low-density lipoprotein cholesterol (LDL-C), or lower levels of high-density lipoprotein cholesterol (HDL-C), which has become a significant public health problem in global (1). The prevalence of high TC, high LDL-C, low HDL-C, and high TG in Chinese adults aged 35 to 75 was 7.1, 4.0, 15.6, and 16.9%, respectively (2). Moreover, ischemic heart disease and stroke are closely related to dyslipidemia, which accounts for over one-third of fatalities associated with these conditions (1). Thus, it is imperative to investigate the risk factors of dyslipidemia to prevent and treat it at an early stage.

In recent years, growing evidence has suggested that environmental metal exposures, including nonessential and essential metals, are associated with dyslipidemia (3, 4). Lead (Pb) is a nonessential toxic metal considered an environmental endocrine disruptor. Chinese are still exposed to low levels of Pb, despite government controls on Pb pollution (5, 6). Our previous studies have demonstrated that the blood Pb level was positively associated with fasting plasma glucose level, the prevalence of metabolic fatty liver disease, and diabetic complications (7–9). However, limited studies have investigated the associations between blood Pb and serum lipid profiles. In addition, the associations of essential metals with serum lipid profiles found in previous studies were inconsistent. For instance, a negative correlation was observed between serum magnesium (Mg) and lipid profiles (TC, LDL-C, and TG) among patients with diabetes (3). However, another study reported a positive association of plasma Mg with TC and LDL-C among Mediterranean adults (10).

Generally, humans are exposed to a variety of metals simultaneously. Mixtures of metals were considered to have different effects on human health than a single metal, since multiple metals may interact synergistically, antagonistically, or in other ways (11, 12). The association of metal exposure with serum lipids was not identical when different exposure profiles were considered (13). One cohort study demonstrated that the metal mixture, including Pb, aluminum, arsenic, barium, vanadium, and zinc (Zn), was associated with an increased risk of incident dyslipidemia (14). However, one recent cohort study with 573 manganese (Mn)-exposed workers did not find a significant cumulative effect of the metal mixture, including Pb and the other nine metals, on changes for TC, TG, or LDL-C (15). Currently, more and more Chinese supplement multivitamin tablets, which include various essential metals; however, the studies investigated the associations between a metal mixture including Pb and essential metals that are often tested clinically and the lipid profiles were limited. Moreover, the potential interactions of Pb and essential metals with lipid profiles have yet to be studied.

Thus, considering the detection frequency of essential metals in clinical work, we finally selected six essential metals in the current study, including Mg, Mn, copper (Cu), iron (Fe), Zn, and calcium (Ca). The current study aimed to examine the associations of a metal mixture including Pb and essential metals including Mg, Mn, Cu, Fe, Zn, and Ca with serum lipid profiles in Chinese community-dwelling adults. Furthermore, we also investigated the potential non-linear exposure-response relationship of Pb and essential metals with serum lipid profiles and the interactions among the metals.

## Methods

### Study design and population enrollment

Participants were enrolled from Lecong, Shunde District, Foshan, China, in 2021. The criteria for inclusion included being over 18 years old and not pregnant, as well as living in Shunde for at least 6 months. Among 1,111 potential participants, 4 were excluded for lack of blood metal data and 109 for taking lipid-lowering drugs. As a result, this study included 998 participants (Supplementary Figure 1).

The current study has been registered at^1^ (ChiCTR2100054130). Based on the 1975 Helsinki Declaration, the Ethics Committee of Shunde Hospital of Southern Medical University approved the study protocol (20211103). Consent was obtained from all participants in an informed and written manner.

### Measurements

A standard questionnaire was administered by trained study personnel to collect sociodemographic characteristics, lifestyle characteristics, and medication information from participants (16). We measured the height and weight of the subjects according to a standard protocol, and body mass index (BMI) was calculated by dividing weight in kilograms by height in meters squared (kg/m^2^) (17). Blood pressure was measured by an automated electronic device (HEM-752 FUZZY, Omron, China) on the nondominant arm twice with at least a 1-min interval following a 5-min rest (18). We calculated the average systolic and diastolic blood pressure based on these two readings.

The fasting blood samples were collected from all participants from 08:00 to 10:00 after an overnight fast of at least 10 h. The whole blood samples were collected in vacuum tubes containing heparin sodium and used to measure blood Pb and the essential metal levels by a quadrupole inductively coupled plasma mass spectrometer (ICP-MS) equipped with a concentric glass nebulizer and a cyclonic spray chamber (7,700x ICP-MS system, Agilent Technologies, CA, USA). A total of 200 μL blood samples were diluted 1:20 (v/v) with a solution containing 0.1% triton X-100 (Sigma-Aldrich, France) and 0.1% nitric acid (69%, Merck, Germany). ICP-MS was used to quantify the diluted samples after vortexing them in a table-top vortexer for a minute. The detailed ICP-MS operating conditions and analysis are consistent with the previous study (19). The performances of the ICP-MS methods, including a low limit of detection (LoD), low limit of quantification (LoQ), accuracy, and precision, were provided in the supplementary materials.

Serum lipid profiles (including TC, TG, HDL-C, and LDL-C) and plasma glucose levels were conducted by BS800 (Mindray, Shenzhen, China). The blood samples for the fasting plasma glucose (FPG) and 2-h postprandial plasma glucose (PPG) levels after carrying out an oral 75 g glucose tolerance test were collected into vacuum tubes with the anticoagulant sodium fluoride. Among people with self-reported diabetes, only fasting plasma glucose and HbA1c were measured. Glycated hemoglobin (HbA1c) was assessed by high-performance liquid chromatography (HLC-723G8, TOSOH, Japan). All the samples were tested (including blood metal, serum lipid profiles, etc.) in Da-An Clinical Laboratory Center in Foshan, Guangdong, a certified laboratory by the College of American Pathologists, within 2 h under cold chain management. Moreover, all the testing items participated in the External Quality Assessment (EQA) of the National Center for Clinical Laboratories and achieved good results.

### Outcome definitions

We categorized the ages into three categories (≤40, 41–60, and > 60 years) as the previous study (20). Education was categorized into three levels: below high school, high school, and beyond high school. Smoking status was classified as non-smokers (past consumption of fewer than 100 cigarettes), former smokers (over 100 cigarettes consumed in the past and more than six months without smoking), and current smokers (past consumption of over 100 cigarettes and smoking currently or within six months) (21). BMI categories were defined as obesity (BMI ≥28 kg/m^2^), overweight (BMI ≥24, <28 kg/m^2^), and normal weight (BMI <24 kg/m^2^) according to Chinese criteria (22). Alcohol consumption was reported as standard drinks and converted to grams by multiplying by 14, and abused drink was defined as >30 g/day for men and > 20 g/day for women (23). The definition of hypertension was systolic blood pressure ≥ 140 mmHg or diastolic blood pressure ≥ 90 mmHg, and/or self-reported previous diagnosis of hypertension by physicians (24). Diabetes was defined as FPG level ≥ 7.0 mmol/L, PPG ≥11.1 mmol/L, or HbA1c ≥6.5% and/or having a self-reported diagnosis of diabetes as the previous study (25). Higher TC (≥ 6.22 mmol/L), higher TG (≥ 2.26 mmol/L), higher LDL (≥ 4.14 mmol/L), and lower HDL (< 1.04 mmol/L) were defined as before (16).

### Statistical analysis

The baseline characteristics of participants were summarized as mean ± standard deviation for continuous variables and frequencies for categorical variables (%) according to sex. Data of TG, blood Pb, and essential metal concentrations were transformed into a natural logarithm for further analysis.

Using Pearson’s correlation analysis, the relationships between blood metal concentrations were classified as strong (*r* > 0.8), medium (> 0.3), and weak (*r* ≤ 0.3) according to the correlation coefficients (*r*) (26). The multivariable linear regression, weighted quantile sum (WQS) regression, and Bayesian kernel machine regression (BKMR) models were used to identify the associations of the blood metals with the serum lipid profiles. To further validate the potential non-linear relationships of Pb and lipid profiles, restricted cubic spline (RCS) analysis with three knots was performed (at the 10th, 50th, and 90th percentiles).

The multivariable linear regression: blood metal levels were grouped as quartiles to assess potential linear or non-linear associations, with the lowest quartile as the reference group. The multivariable linear regression was performed to evaluate the associations of quartiles of Pb and essential metals with TC, LDL, HDL, and Ln TG, considering multiple-metal analysis (including all metals simultaneously). Age, sex, education, smoking status, abused drink, BMI categories, hypertension, and diabetes were adjusted in the full model of all the analyses. Covariates were tested for collinearity based on the variance inflation factor (VIF) < 5 (27).

WQS regression: We split the data into a training dataset and a testing dataset (40:60), with 1,000 boot-strap samplings in WQS, where variable weights were estimated on the training dataset, and mixture significance was determined on the testing dataset (28, 29). Metals with estimated weights greater than 0.143 (1/7) were considered to have a significant impact on the score of the WQS, which is calculated by dividing the sum of the absolute values of the weights by the total number of metals in the mixture (28, 29). Both positive and negative WQS scores were implemented and evaluated. The R package gWQS was used.

BKMR model: The BKMR model was used to identify the associations of the mixture of Pb and the essential metals on the serum lipid profiles. The posterior inclusion probabilities (PIPs) for each metal and the total influence of the metal mixture on the serum lipid profiles were calculated after fitting the final model using the Markov Chain Monte Carlo (MCMC) sampler for 50,000 iterations (30). A PIP threshold of 0.5 is typically used to determine if the investigated heavy metal is important (24, 31). We also displayed the dose–response curves for each metal and the potential interactions between the metals based on predicted serum lipid profiles when holding all other metals exposure at the median, 10th, or 90th percentile using the BKMR model as previously described (13). The R package bkmr was used.

All data were analyzed using IBM SPSS Statistics (version 27) and R (version 4.2.1). A two-tailed *p* value <0.05 was considered statistically significant.

## Results

### General characteristics of participants

Table 1 describes the basic characteristics of the participants. The mean age of the overall study population was 48.6 ± 14.13 years, and 42.7% were men. The prevalence of overweight or obesity, diabetes, and hypertension in the population was 43.1, 13.3, and 32.1%, respectively. The prevalence of higher TC, higher TG, higher LDL, and lower HDL was 21.3, 13.1, 14.9, and 9.4%, respectively. The mean level of blood Mg, Mn, Ca, Fe, Cu, Zn, and Pb was 41.66 mg/L, 13.13 μg/L, 62.91 mg/L, 505.86 mg/L, 884.65 μg/L, 6.32 mg/L, and 20.00 μg/L, respectively.

**Table 1:** General Characteristics of participants in the study.

	Total (*n* = 998)	Men (*n* = 426)	Women (*n* = 572)
Age, years	48.59 ± 14.13	47.36 ± 14.19	49.50 ± 14.03
Education, %			
< high school	27.0	22.5	30.2
high school	35.0	35.2	34.8
> high school	38.1	42.3	35.0
BMI, %			
Normal weight	56.9	50.2	61.9
Overweight	31.1	33.3	29.4
Obese	12.0	16.4	8.7
Smoking status, %			
No	81.1	55.9	99.8
Ever	4.7	10.8	0.2
Current	14.2	33.3	0.0
Abuse drinking, %	1.7	4.0	0.0
Hypertension, %	32.1	38.7	27.1
Diabetes, %	13.3	13.4	13.3
TC, mmol/L	5.43 ± 1.11	5.43 ± 1.13	5.42 ± 1.10
HDL-C, mmol/L	1.42 ± 0.32	1.31 ± 0.31	1.49 ± 0.31
LDL-C, mmol/L	3.23 ± 0.92	3.37 ± 0.94	3.12 ± 0.89
TG, mmol/L	1.19 ± 0.87	1.37 ± 1.10	1.10 ± 0.67
FPG, mmol/L	5.02 ± 0.73	5.04 ± 0.78	5.02 ± 0.71
HbA1c, %	5.70 ± 0.60	5.70 ± 0.60	5.60 ± 0.67
Mg, mg/L	41.66 ± 4.49	43.26 ± 4.05	40.48 ± 4.43
Mn, μg/L	13.13 ± 4.02	12.24 ± 3.49	13.79 ± 4.25
Ca, mg/L	62.91 ± 5.75	60.45 ± 5.51	64.74 ± 5.22
Fe, mg/L	505.86 ± 57.43	542.94 ± 46.15	478.25 ± 48.82
Cu, μg/L	884.65 ± 121.29	837.70 ± 107.81	919.61 ± 119.03
Zn, mg/L	6.32 ± 0.92	6.67 ± 0.84	6.06 ± 0.88
Pb, μg/L	20.00 ± 12.00	23.00 ± 11.00	18.00 ± 11.00

The baseline characteristics of participants were summarized as mean ± standard deviation for continuous variables, and frequencies for categorical variables (%).

TC, total cholesterol; TG, triglyceride; HDL-C, high-density lipoproteins cholesterol; LDL-C, low-density lipoproteins cholesterol; FPG, fasting plasma glucose; HbA1c, glycated hemoglobin; BMI, body mass index; Mg, magnesium; Mn, manganese; Ca, calcium; Fe, iron; Cu, copper; Zn, zinc; Pb, lead.

### The associations of blood Pb and essential metal levels with serum lipid profiles using the multivariable linear regression

Using Pearson’s correlation analysis, weak-to-moderate correlations between blood metals were observed (*r*: 0.02 to 0.70; Supplementary Figure 2). No significant collinearity of the covariates, including the metals, were found (all VIF < 3) (Supplementary Table 1).

**Figure 1:**
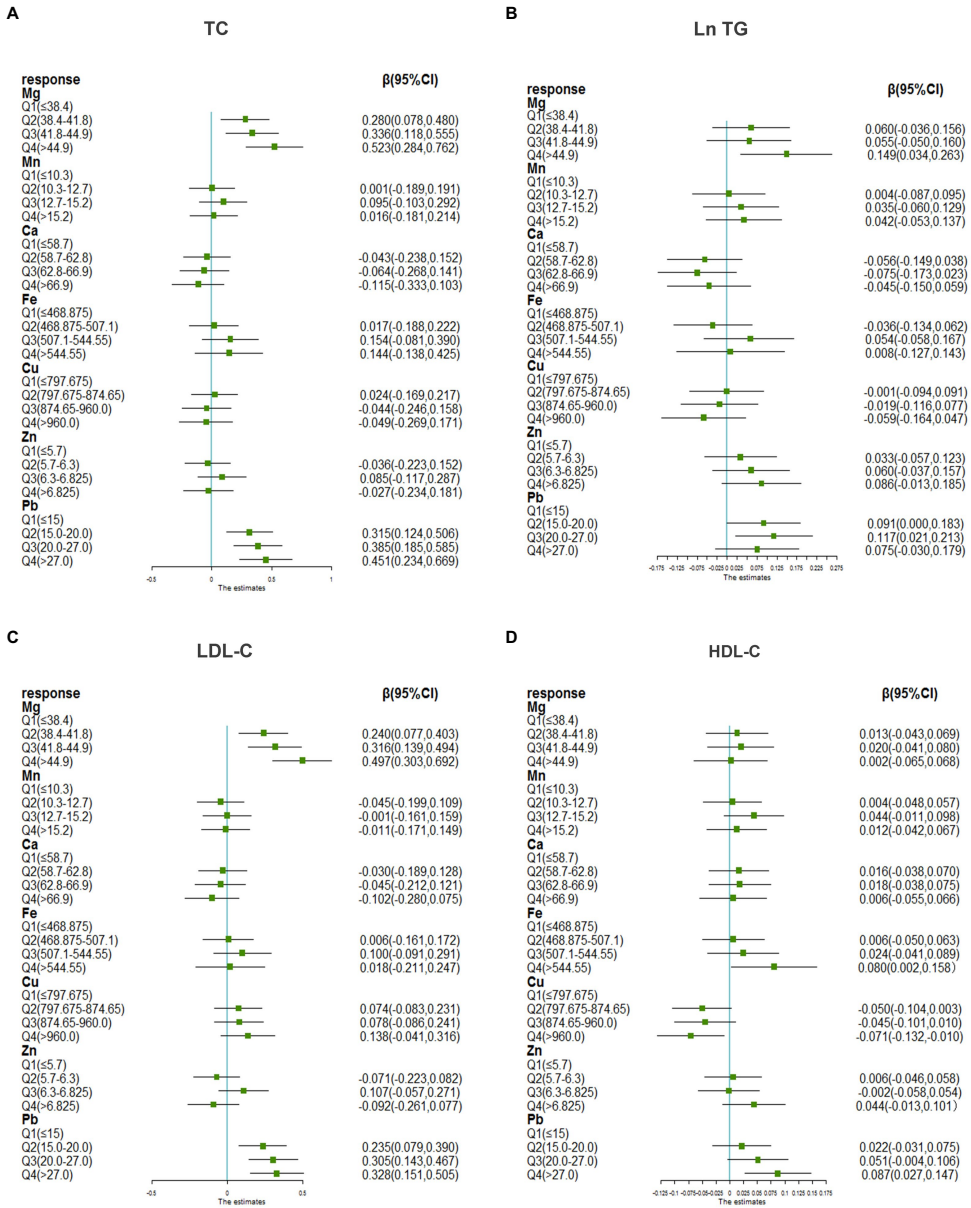
The associations of blood Pb and essential metals with serum lipid profiles using multivariable liner regression. **(A)** Metals and TC, **(B)** metals and Ln TG, **(C)** metals and LDL-C, **(D)** metals and HDL-C. TG was transformed to natural logarithm and metal concentrations were grouped into quartiles for further analysis. The model was adjusted for age, sex, educational, smoking status, abuse drinking, BMI categories, diabetes, and hypertension. TC, total cholesterol; TG, triglyceride; LDL-C, low-density lipoproteins cholesterol; HDL-C, high-density lipoproteins cholesterol; BMI, body mass index; Mg, magnesium; Mn, manganese; Ca, calcium; Fe, iron; Cu, copper; Zn, zinc; Pb, lead.

Figure 1 presents the associations of metals with TC, Ln TG, LDL-C, and HDL-C using the multivariable linear regression model considering multiple-metal analysis. Compared with the lowest quartile, individuals in the highest quartile of Mg had the highest *β* for TC [0.523 (0.284, 0.762)], Ln TG [0.149 (0.034, 0.263)], and LDL-C [0.497 (0.303, 0.692)] (*p* < 0.05, all). Compared with the lowest quartile, individuals in the highest quartile of Fe had the highest *β* [0.080 (0.002, 0.158)] (*p* < 0.05) for HDL. However, individuals in the highest quartile of Cu had the lowest *β* [−0.071 (−0.132, −0.010)] (*p* < 0.05). Compared with the lowest quartile, individuals in the highest quartile of Pb had the highest *β* for TC [0.451 (0.234, 0.669)], LDL-C [0.328 (0.151, 0.505)], and HDL-C [0.087 (0.027, 0.147)] (all the *p* < 0.05), while individuals in the third quartile of Pb had the highest *β* [0.117 (0.021, 0.213)] (*p* < 0.05) for Ln TG. The interactions between Pb and Mg on TC (*p* for interaction = 0.068) and LDL-C (*p* for interaction = 0.046) were found.

### The associations of the mixture of blood Pb and essential metals with serum lipid profiles using the WQS model

**Figure 2:**
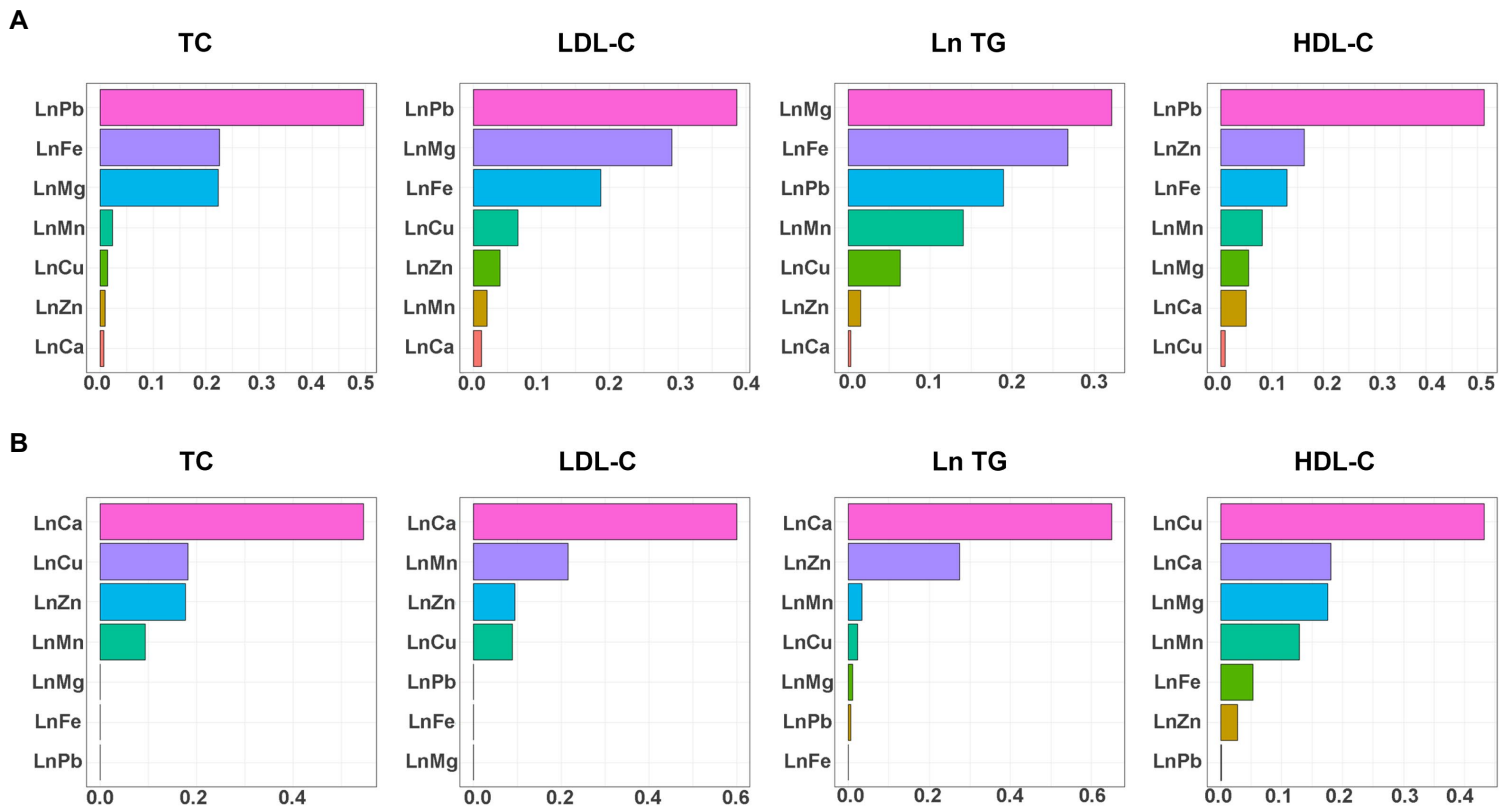
WQS regression weights of blood Pb and essential metals for serum lipid profiles. **(A)** The positive direction and **(B)** the negative direction. TG, blood Pb and essential metal concentrations were transformed to natural logarithm for further analysis. The model was adjusted for age, sex, educational, smoking status, abuse drinking, BMI categories, diabetes, and hypertension. TC, total cholesterol; TG, triglyceride; LDL-C, low-density lipoproteins cholesterol; HDL-C, high-density lipoproteins cholesterol; BMI, body mass index; Mg, magnesium; Mn, manganese; Ca, calcium; Fe, iron; Cu, copper; Zn, zinc; Pb, lead.

Table 2 presents the associations of mixed metals on lipid profiles using the WQS model. The positive WQS indices of the metal mixture were associated with all the serum lipid profiles (all the *p* < 0.05), suggesting that the metal mixture was positively associated with lipid profiles. In the positive direction, blood Pb received the highest weights for TC (49.6%), LDL-C (38.6%), and HDL-C (51.3%), and blood Mg (32.1%) received the highest weights for Ln TG (Figure 2). In the negative direction, blood Ca received the highest weights for TC (54.6%), LDL-C (60.0%), Ln TG (64.9%), and blood Cu received the highest weights for HDL-C (43.2%) (Figure 2).

**Table 2:** Associations of the mixture of blood Pb and the essential metals with serum lipids profiles.

	Positive WQS regression	*p*	Negative WQS regression	*p*
TC	0.394 (0.265, 0.523)	<0.001	0.023 (−0.108, 0.154)	0.728
Ln TG	0.080 (0.015, 0.145)	0.016	0.019 (−0.038, 0.076)	0.510
LDL-C	0.369 (0.256, 0.482)	<0.001	0.060 (−0.044, 0.164)	0.263
HDL-C	0.071 (0.034, 0.108)	<0.001	0.013 (−0.024, 0.050)	0.503

Data are presented as the WQS regression index (95% CI), representing the overall mixture effect on serum lipids profiles. The model was adjusted for age, sex, education, smoking status, abused drink, BMI categories, hypertension and diabetes.

TC, total cholesterol; TG, triglyceride; LDL-C, low-density lipoprotein cholesterol; HDL-C, high-density lipoprotein cholesterol; WQS, weighted quantile sum.

### The associations of the mixture of blood Pb and the essential metals with serum lipid profiles using the BKMR model

**Figure 3:**
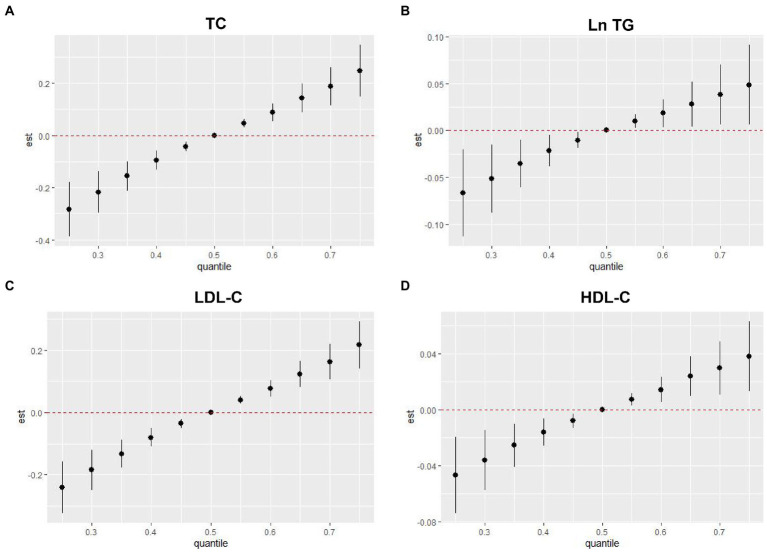
Overall associations of metal mixture including blood Pb and essential metals with serum lipid profiles in the BKMR model. **(A)** Metal mixture and TC, **(B)** metal mixture and Ln TG, **(C)** metal mixture and LDL-C, and **(D)** metal mixture and HDL-C. TG, blood Pb and essential metal concentrations were transformed to natural logarithm for further analysis. The model was adjusted for age, sex, educational, smoking status, abuse drinking, BMI categories, diabetes, and hypertension. Compared with the 50th percentile, the estimate at any percentile where the 95% confidence interval does not include 0 is considered statistically significant. TC, total cholesterol; TG, triglyceride; LDL-C, low-density lipoproteins cholesterol; HDL-C, high-density lipoproteins cholesterol; BMI, body mass index; Mg, magnesium; Mn, manganese; Ca, calcium; Fe, iron; Cu, copper; Zn, zinc; Pb, lead.

**Figure 4:**
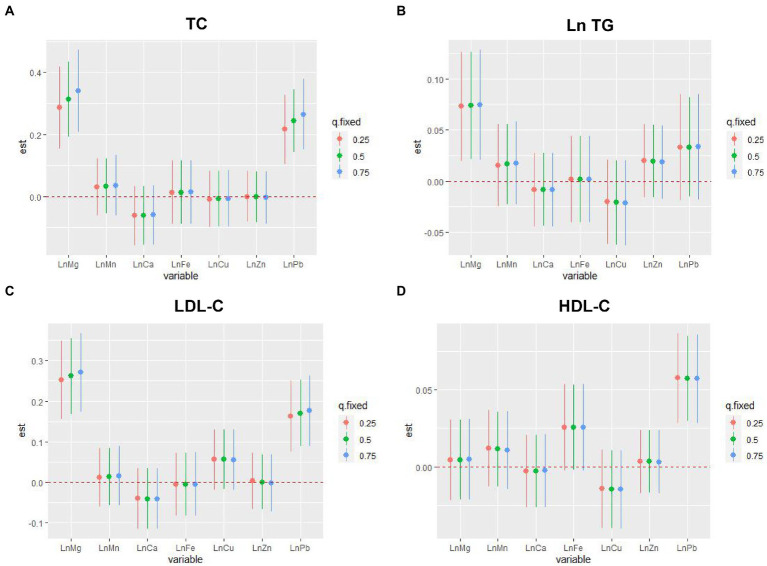
The single-exposure associations of individual metal with serum lipid profiles by the BKMR model. **(A)** Metal mixture and TC, **(B)** metal mixture and Ln TG, **(C)** metal mixture and LDL-C, and **(D)** metal mixture and HDL-C. TG, blood Pb and essential metal concentrations were transformed to natural logarithm for further analysis. The model was adjusted for age, sex, educational, smoking status, abuse drinking, BMI categories, diabetes, and hypertension. The associations were analyzed when all the other exposures were fixed to 25th, 50th, and 75th percentile. The 95% confidence interval of the estimate does not include 0 is considered statistically significant. TC, total cholesterol; TG, triglyceride; LDL-C, low-density lipoproteins cholesterol; HDL-C, high-density lipoproteins cholesterol; BMI, body mass index; Mg, magnesium; Mn, manganese; Ca, calcium; Fe, iron; Cu, copper; Zn, zinc; Pb, lead.

**Figure 5:**
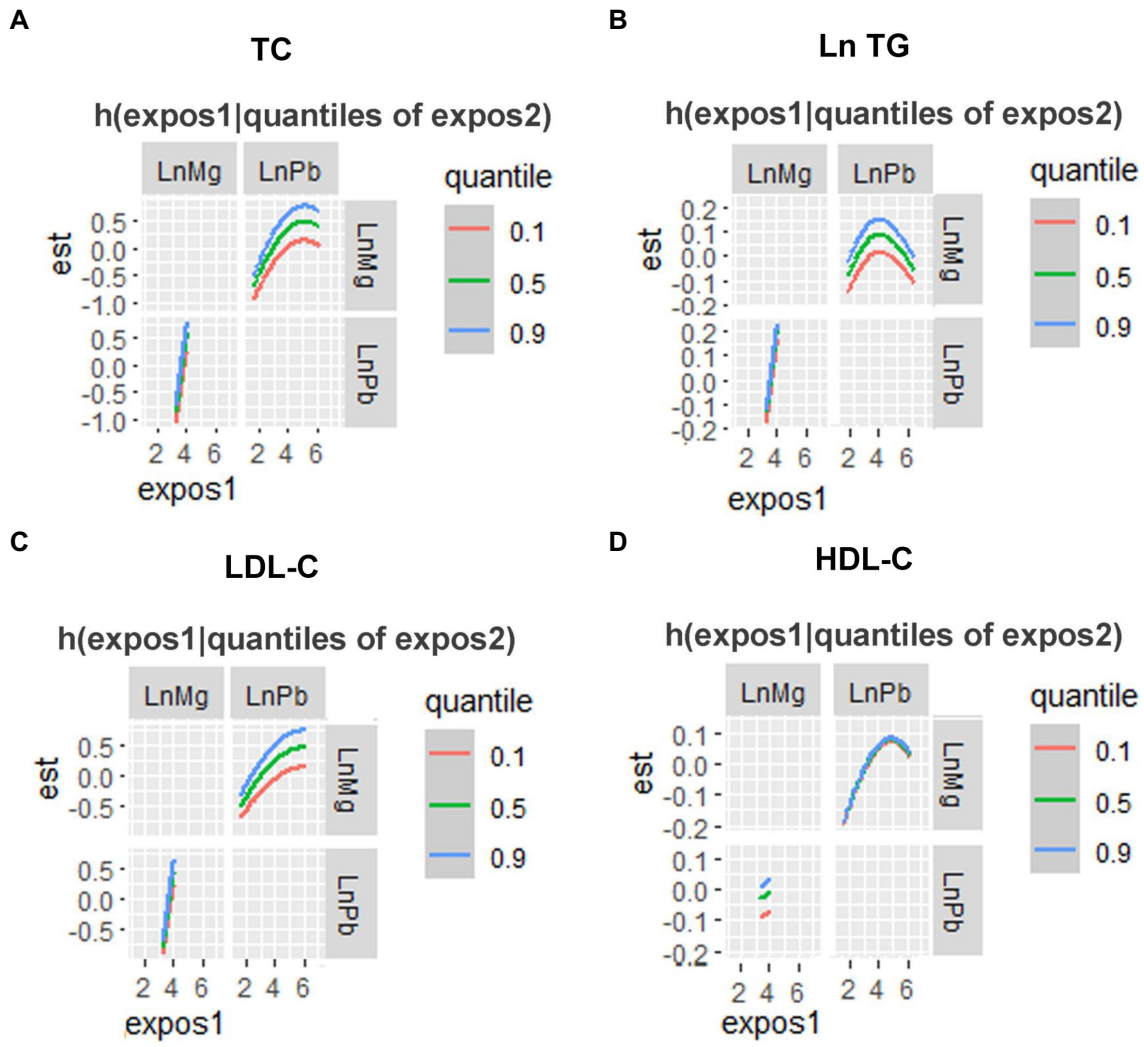
The bivariate associations of Mg and Pb exposure-response relationships of a single metal where the second element was fixed at P10, P50 and P90 analyzed by the BKMR model. **(A)** TC, **(B)** Ln TG, **(C)** LDL-C, and **(D)** HDL-C. TG, blood Pb and essential metal concentrations were transformed to natural logarithm for further analysis. The model was adjusted for age, sex, educational, smoking status, abuse drinking, BMI categories, diabetes, and hypertension. TC, total cholesterol; TG, triglyceride; LDL-C, low-density lipoproteins cholesterol; HDL-C, high-density lipoproteins cholesterol; BMI, body mass index; Mg, magnesium; Pb, lead.

In the BKMR model, as the metal mixture approached and exceeded the 55th percentile, the TC, Ln TG, LDL-C, and HDL-C increased significantly (Figure 3). When other elements were fixed at their 25th, 50th, or 75th percentile, the associations of Mg and Pb with TC and LDL-C, the association of Mg with Ln TG, and the association of Pb with HDL-C were significant (Figure 4). Figure 5 presents the bivariate Mg and Pb exposure-response relationships. The trend of the curves of Pb on TC and LDL-C intersected when Mg was at the 10th, 50th, or 90th percentile, which suggested the interactions between Pb and Mg on TC and LDL-C. No interactions among other metals were found on the lipid profiles. In addition, an inverse U-shaped association of Pb with Ln TG was found. The non-linear relationship between Pb and Ln TG was further validated using RCS analysis (*p* for overall = 0.030, *p* for non-linear = 0.024) (Supplementary Figure 3). Supplementary Table 2 shows the important metals on lipid profiles: on TC and Ln TG, Mg was considered the most important metal (both PIPs >0.9); on LDL-C, Mg and Pb were identified as the most two important metals (both PIPs >0.9); on HDL-C, Pb was recognized as the most important metal (PIP > 0.9).

## Discussion

In the current study, we examined the associations of the mixture, including blood Pb and the essential metals, with serum lipids profiles using a variety of statistical approaches. The linear regression pointed out that higher blood Pb levels were associated with higher serum TC, LDL-C, and HDL-C levels; higher blood Mg levels were associated with higher serum TC, LDL-C, and Ln TG levels. In addition, our findings were mainly robust in BKMR and WQS regression. The metal mixture, including blood Pb and essential metals, was positively associated with the serum lipid profiles, including TC, Ln TG, LDL-C, and HDL-C. An inverse U-shaped association of Pb with Ln TG and the positive interaction between blood Pb and Mg levels on TC and LDL-C were also found. The findings implied that supplementation of multivitamin tablets should be cautious in people with low doses of Pb exposure, considering the increased risk of dyslipidemia, which could contribute to the prevention strategies for dyslipidemia.

Although most of the results in the current study seemed reliable and robust, the significantly important metals identified by these approaches are not exactly consistent. For example, blood Fe level is not significantly associated with LDL-C in the linear regression; however, WQS regression and BKMR suggested Fe was an important metal for increasing LDL-C. The inconsistency of the results can be attributed to the pros and cons of each approach. Multivariable linear regression is typically used, as its results are straightforward to interpret. However, combining metals with high correlation in the linear regression model is not recommended since it may distort the results (32). We conducted the linear regression considering multiple-metal analysis in the current study because the metals were only with weak-to-moderate correlations. By comparison, BKMR or WQS regression can include multiple metals with moderate to high correlations (30). However, mixture exposure burden and outcomes can only be examined in one direction per occasion using the WQS model (29). Although BKMR can capture the important metals in either direction or their non-linear exposure-response relationships, this method cannot determine co-exposure patterns of metals at both high and low concentrations (30). Thus, to estimate the single and combined associations of blood metals with the outcome, various statistical methods should be used, and the results interpreted together, weighing their advantages and disadvantages (30, 33).

The associations of Pb and lipid profiles determined in previous studies were inconsistent. One study (the mean of Pb is 28.07 μg/L) found that Pb levels were positively associated with the prevalence of dyslipidemia among 1,013 elders (34). Another study (the mean of Pb is 12.3 μg/L) enrolled general adults found that higher Pb was associated with elevated TC and LDL-C but not with TG (35). One recent study reported that elevated HDL-C is associated with lower blood Pb in Pb-exposed workers (the mean of Pb is 139.94 μg/L) (36). In the current study, we found that blood Pb, with relatively large weights in the metal mixture, was positively associated with TC, LDL-C, and HDL-C while non-linearly associated with Ln TG. The differences may be due to the different blood Pb levels of the participants in these studies and the non-linear relationship of Pb with TG. In animal experiments, similar conclusions were given (37, 38). Mouse exposed to Pb (5 mg/kg body weight) for 30 days orally caused higher TC, LDL-C, HDL-C, and lower TG (37). The inverse U-shaped association of Pb with TG was also observed in the Wistar rats (38).

In previous studies, conflicting results have been found regarding Mg and lipid profiles (39). One study with 492 participants reported a significantly positive association of Mg with TC and LDL-C (10), which is consistent with our results. The positive associations of Mg with TC, LDL-C, and TG were also found in two earlier studies (40, 41). However, in two other studies enrolling patients with diabetes or chronic kidney disease, negative associations of Mg with TC, LDL-C, and TG were found (3, 42). Furthermore, studies about the efficacy of Mg supplementation on decreasing serum lipids are conflicting (39). One earlier meta-analysis study suggested no significant effects of Mg supplementation on the lipid profiles among individuals with or without diabetes (43). However, one recent meta-analysis study concluded that Mg supplementation could significantly lower the LDL level in patients with diabetes (44). These different findings may be accounted for the blood Mg levels of the population enrolled in our study being higher than those of patients with diabetes, chronic kidney disease, or hypomagnesemia (3, 42, 44). It indicates that overindulging or unnecessary Mg supplementation may harm lipid profiles. Another possible reason may be the simple-binding interaction between Mg and lipoprotein particles (40). The affinity of certain phospholipid head groups to Mg was given as a divalent cation, causing a positive correlation between Mg and all lipoprotein species (41).

As far as we know, no studies have investigated the relationships of a metal mixture, including Pb and essential metals, with lipid profiles, although previous studies found that the metal mixture was closely linked to diabetes (11), non-alcoholic fatty liver diseases (31, 45) and all-cause mortality (46). Using the BKMR and WQS regression models, we found that the blood metal mixture was positively associated with all of the lipid profiles. Interestingly, positive interactions between Pb and Mg on TC and LDL-C were also found. Scarce previous studies have reported the interaction between Pb and Mg on human health. It has been demonstrated that Pb can increase the production of cholesterol, leading to hypercholesterolemia by the upregulation of HMG-CoA reductase, an enzyme involved in the biosynthesis of cholesterol (47), and Mg also plays a vital role in the rate-limiting step in cholesterol synthesis at HMG-Co A reductase (40). In addition, sub-chronic lead exposure had no effects on Mg levels in any of the analyzed tissues (48), and Mg even could alleviate the adverse effects of Pb (41). Thus, we speculated that Pb might promote the binding of Mg to lipoprotein particles and further reduce the biological function of Mg. Nevertheless, future studies are needed to elucidate the interaction mechanism of Pb and Mg on lipid metabolism.

The current study still has several limitations, although it has some strengths, such as utilizing diverse statistical approaches to identify the associations of metals with lipid profiles. Firstly, causal inference cannot be drawn due to the study’s cross-sectional nature. Further cohort studies and vivo experiments are needed to investigate the possible causal relationship. Secondly, despite our best efforts to adjust for the potential covariates, not all confounders or metals were measured in the current study. Thirdly, since this study included only Han Chinese participants and the number of participants was relatively small, the results may not apply to people of other ethnicities. Further studies with larger sample sizes are needed.

In conclusion, the positive associations of the metal mixture, including blood Pb and essential metals with serum lipid profiles, and the positive interactions between Pb and Mg on TC and LDL-C were observed. The levels of blood Pb, together with essential metals, especially Mg levels, are suggested to be considered when assessing dyslipidemia risk. However, more evidence from prospective cohort studies with larger sample sizes and mechanism researches are still needed to validate the conclusions.

## Data availability statement

The raw data supporting the conclusions of this article will be made available by the authors, without undue reservation.

## Ethics statement

The Ethics Committee of Shunde Hospital of Southern Medical University approved the study protocol (20211103). The patients/participants provided their written informed consent to participate in this study.

## Author contributions

HW and LL performed the conceptualization. HW and DW conducted the data analysis. YaH, TL, YiH, HG, JW, ZL, YL, and XL conducted the data acquisition. HW drafted the manuscript. JS revised the manuscript and served as scientific advisors. All authors contributed to the article and approved the submitted version.

## Funding

This work was supported by the National Natural Science Foundation of China (82200960 and 82170800) and the Guangdong Basic and Applied Basic Research Foundation (2021A1515110682).

## Conflict of interest

The authors declare that the research was conducted in the absence of any commercial or financial relationships that could be construed as a potential conflict of interest.

## Publisher’s note

All claims expressed in this article are solely those of the authors and do not necessarily represent those of their affiliated organizations, or those of the publisher, the editors and the reviewers. Any product that may be evaluated in this article, or claim that may be made by its manufacturer, is not guaranteed or endorsed by the publisher.

## Footnotes

^1^ www.chictr.org.cn
