# Supplementary material for: Single and combined associations of blood lead and essential metals with serum lipid profiles in community-dwelling adults
Source: Front Nutr. 2023 Apr 14;10:1129169. doi: 10.3389/fnut.2023.1129169 (PMC10140323; doi:10.3389/fnut.2023.1129169)
Supplement: Supplementary file 1 [file Data_Sheet_1.docx]

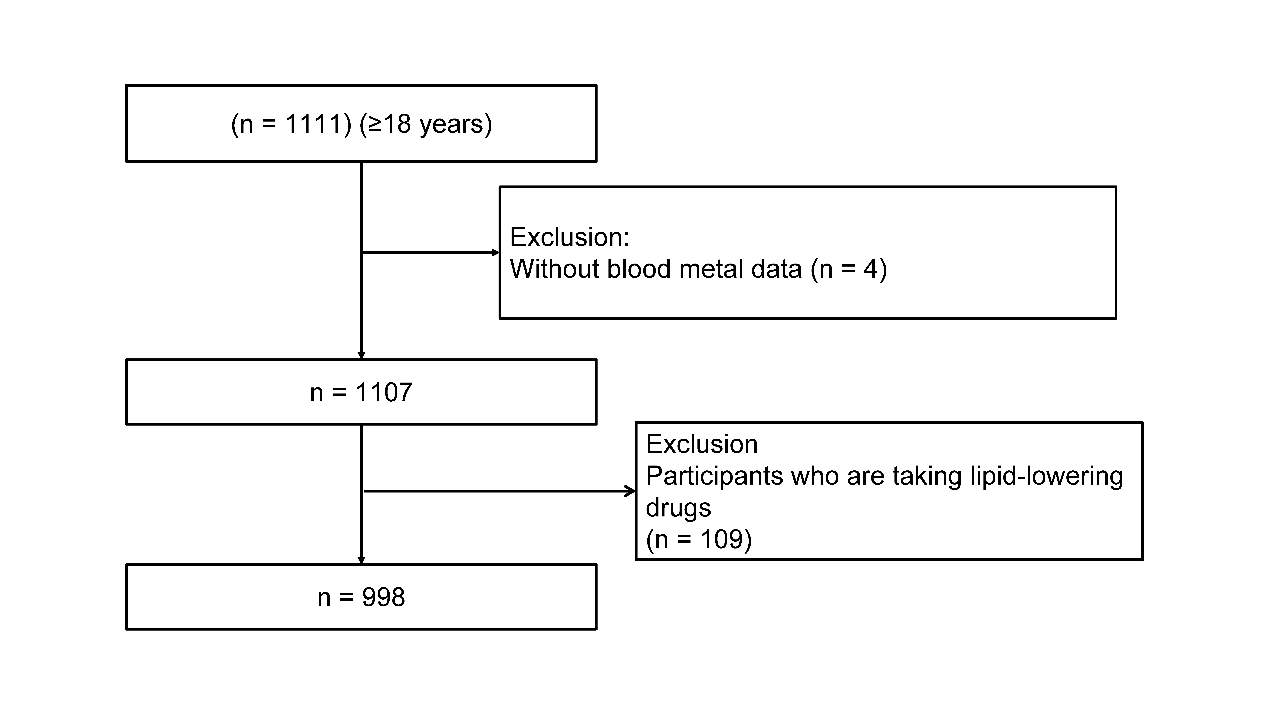


**Supplementary Figure 1.** Flow chart of sample pooling and final sample used for the analysis.


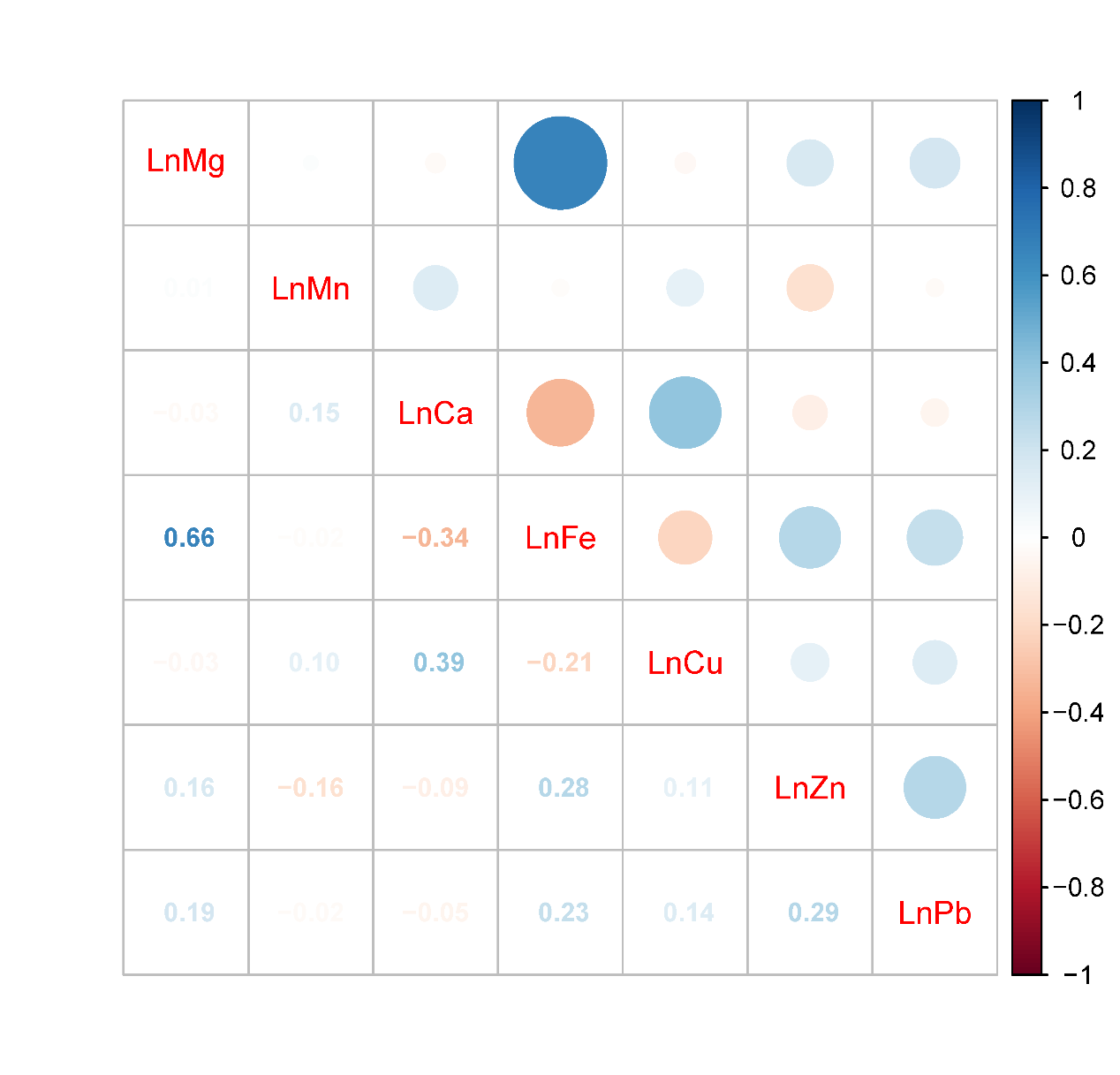


**Supplementary Figure 2.** The Correlations between different metal concentrations.

Blood Pb and essential metal concentrations were transformed into natural logarithm (Ln) for further analysis. Pearson’s correlation coefficients were determined between Ln Pb and Ln essential trace element concentrations. The bigger and darker color circle represents higher levels of correlation coefficients.


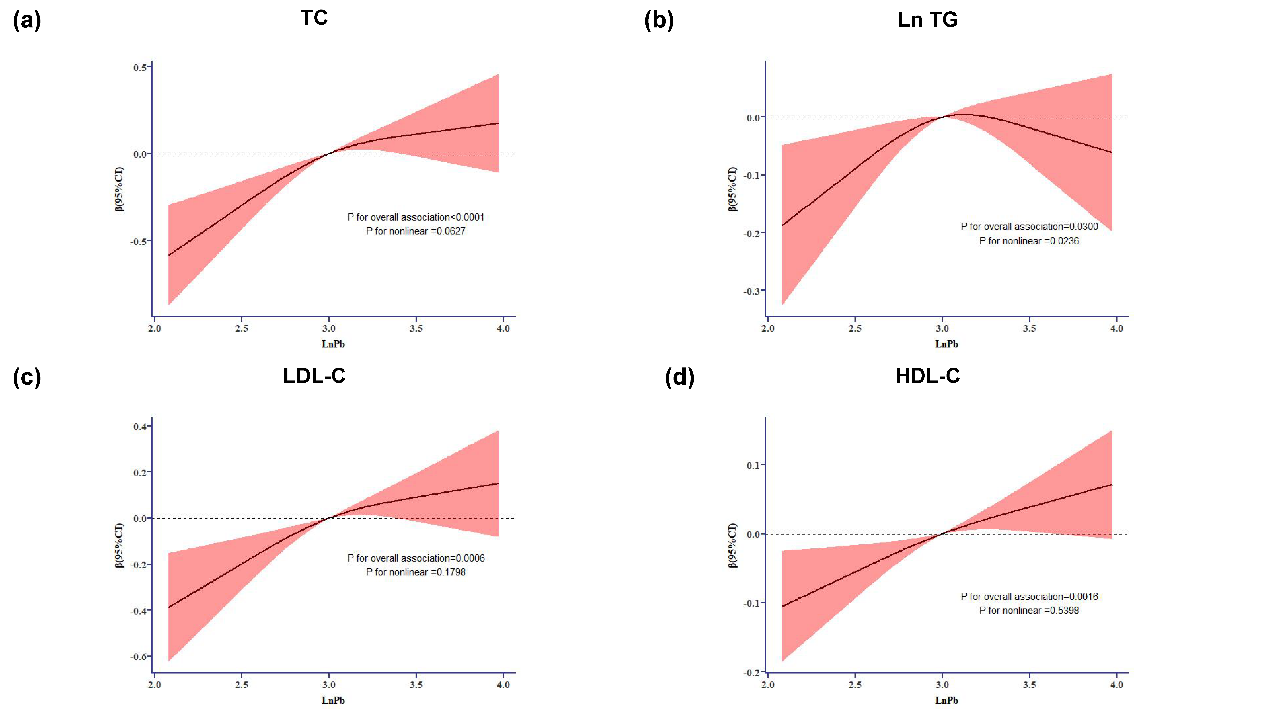


**Supplementary Figure 3**. The dose-response analysis of blood Pb with serum lipid profiles.

(a) Pb and TC (b) Pb and Ln TG (c) Pb and LDL-C (d) Pb and HDL-C

The middle line and upper and lower line represent the β and its 95% confidence interval, respectively. RCS analysis was used. The model was adjusted for age, sex, educational, smoking status, abuse drinking, BMI categories, diabetes and hypertension.

TC, total cholesterol; TG, triglyceride; LDL-C, low-density lipoproteins cholesterol; HDL-C, high-density lipoproteins cholesterol; BMI, body mass index; Pb, lead; RCS, restricted cubic spline.

**Supplementary table 1.** The collinearity diagnosis of the covariates

| Characteristic | VIF |
| --- | --- |
| Age | 1.629 |
| Sex | 2.247 |
| Education level | 1.486 |
| BMI categories | 1.13 |
| Smoking status | 1.472 |
| Abused drink | 1.048 |
| Hypertension | 1.197 |
| Diabetes | 1.099 |
| LnMg | 2.006 |
| LnMn | 1.111 |
| LnCa | 1.454 |
| LnFe | 2.672 |
| LnCu | 1.45 |
| LnZn | 1.3 |
| LnPb | 1.4 |

Data are presented as variance infation factor (VIF).

**Supplementary Table 2.** The PIPs of the metals obtained from the BKMR model

| Characteristic | TC | Ln TG | LDL-C | HDL-C |
| --- | --- | --- | --- | --- |
| Ln Mg | 1.000 | 0.939 | 1.000 | 0.543 |
| Ln Mn | 0.478 | 0.356 | 0.396 | 0.494 |
| Ln Ca | 0.656 | 0.504 | 0.592 | 0.524 |
| Ln Fe | 0.578 | 0.537 | 0.515 | 0.832 |
| Ln Cu | 0.459 | 0.497 | 0.615 | 0.553 |
| Ln Zn | 0.497 | 0.525 | 0.500 | 0.453 |
| Ln Pb | 0.100 | 0.526 | 0.995 | 0.990 |

Data are presented as the posterior inclusion probabilities (PIPs).

**Supplementary Table 3.** ICP-MS analytical performance for whole blood

| Metals | LoD | LoQ |
| --- | --- | --- |
| Pb, μg/L | 0.25 | 0.95 |
| Mg, mg/L | 0.01 | 0.03 |
| Mn, μg/L | 0.23 | 0.85 |
| Ca, mg/L | 0.13 | 0.37 |
| Fe, mg/L | 1.5 | 4.3 |
| Cu, μg/L | 0.001 | 0.002 |
| Zn, mg/L | 0.001 | 0.003 |

LoD, low limit of detection; LoQ, low limit of quantification.

The accuracy and precision of the method had been verified by participation in the National Center for Clinical Laboratories (NCCL) external quality assessment (EQA) scheme.
